# Characterization and Expression Analysis of Insulin Growth Factor Binding Proteins (IGFBPs) in Pacific White Shrimp *Litopenaeus vannamei*

**DOI:** 10.3390/ijms22031056

**Published:** 2021-01-21

**Authors:** Ying Pang, Xiaojun Zhang, Jianbo Yuan, Xiaoxi Zhang, Jianhai Xiang, Fuhua Li

**Affiliations:** 1 Key Laboratory of Experimental Marine Biology, Institute of Oceanology, Chinese Academy of Sciences, Qingdao 266071, China; pangying@qdio.ac.cn (Y.P.); yuanjb@qdio.ac.cn (J.Y.); zhangxiaoxi@qdio.ac.cn (X.Z.); jhxiang@qdio.ac.cn (J.X.); fhli@qdio.ac.cn (F.L.); 2 Laboratory for Marine Biology and Biotechnology, Qingdao National Laboratory for Marine Science and Technology, Qingdao 266237, China; 3 College of Earth and Planetary Sciences, University of Chinese Academy of Sciences, Beijing 100049, China; 4 Center for Ocean Mega-Science, Chinese Academy of Sciences, Qingdao 266071, China

**Keywords:** insulin growth factor binding proteins, *Litopenaeus vannamei*, gene structures, gene expression, growth

## Abstract

The insulin signaling (IIS) pathway plays an important role in the metabolism, growth, development, reproduction, and longevity of an organism. As a key member of the IIS pathway, insulin-like growth factor binding proteins (IGFBPs) are widely distributed a family in invertebrates and vertebrates that are critical in various aspects of physiology. As an important mariculture species, the growth of Pacific white shrimp, *Litopenaeus vannamei*, is one of the most concerning characteristics in this area of study. In this study, we identified three IGFBP genes in the genome of *L. vannamei* and analyzed their gene structures, phylogenetics, and expression profiles. *LvIGFBP1* was found to contain three domains (the insulin growth factor binding (IB) domain, the Kazal-type serine proteinase inhibitor (Kazal) domain, and the immunoglobulin C-2 (IGc2) domain), while *LvIGFBP2* and *LvIGFBP3* only contained a single IB domain. *LvIGFBP1* exhibited high expression in most tissues and different developmental stages, while *LvIGFBP2* and *LvIGFBP3* were only slightly expressed in hemocytes. The RNA interference of *LvIGFBP1* resulted in a significantly smaller increment of body weight than that of control groups. These results will improve our understanding of the conservative structure and function of IGFBPs and show potential applications for the growth of shrimp.

## 1. Introduction

Insulin/insulin-like growth factor 1 (IGF-1) signaling (IIS) is critical for an organism’s metabolism, growth, development, reproduction, and longevity related to its nutrient status [1,2,3]. The IIS pathway is highly conserved among invertebrates and vertebrates, and it has shown itself at the stem of the Metazoan clade and maybe in the Choanozoan one [4,5,6]. The biological functions of IGFs, insulin, and insulin-like peptides (ILPs) are mainly regulated by their receptors and insulin-like growth factor binding proteins (IGFBPs) [7]. IGFBPs serve as major regulators of IGF activity by binding and directing the delivery of IGFs to target cells and interacting with IGF receptors (IRs) to regulate downstream signal transduction networks [7,8].

In vertebrates, the IGFBP superfamily has six members (IGFBP1–IGFBP6) and ten related proteins (IGFBP-rP1–IGFBP-rP10); the former proteins can bind IGFs with a high affinity, but the latter ones have poor affinity in binding with IGFs [7,9,10]. IGFBPs contain 240–300 amino acids (22–32 kDa) and have three typical domains: an N-terminal insulin growth factor binding (IB) domain, the mid-region domain (L-domain), and the C-terminal domain (thyroglobulin type I repeat). The mid-region domain is comparatively variable, and the other regions are extremely conserved [11], maintained by disulfide bonds among conserved cysteine residues. There are twelve conserved Cys in the N-terminal IB domain, while the C-terminal domain only possesses six Cys [12]. Previous research has indicated that IGFBP2 and IGFBP3 contain at least two IGF binding determinants: one in the N-terminal IB domain and another in the C-terminal immunoglobulin C-2 (IGc2) domain [12,13,14,15]. In addition, IGFBPs have some potential functions, such as regulating cellular growth and apoptosis [16]. Thus, IGFBPs are critical for many biological processes, including cell proliferation, survival, migration, autophagy, and angiogenesis [17].

In recent years, more than 10 homologues of the IGFBP superfamily have been found in crustacean species, including Eastern rock lobster (*Sagmariasus verreauxi*) [18], red claw crayfish (*Cherax quauricarinatus*) [19], mud crab (*Scylla paramamosain*) [20], and oriental river prawn (*Macrobrachium nipponense*) [21]. In addition, several single IB domain (SIBD) proteins have been reported as novel members of the IGFBP superfamily in the Chinese mitten crab (*Eriocheir sinensis*) [22] and the Pacific white shrimp (*Litopenaeus vannamei*) [11]. Though these IGFBPs, IGFBP-rPs, and SIBDs of invertebrates cannot be classified as typical IGFBP1–6, many of them have been found to bind to insulin or IGFs [7]. Meanwhile, these studies have also shown that the homologues have multiple functions. *C. quauricarinatus* IGFBP (Cq-IGFBP) can modulate the crustacean androgenic hormone and interacts with an insulin-like, gender-specific ligand [19]. *S. verreauxi* IGFBP (Sv-IGFBP) has binding affinities relative to Sv-IAG [18]. *S. paramamosain* IGFBP (Sp-IGFBP-rp1) might be closely involved in ovarian development [20]. *Haliotis diversicolor* IGFBP7 (saIGFBP7) is involved in a function associated with pathogenic infection and may have an impact on the adult abalone immune system [23]. In addition, Yesso scallop (*Patinopecten yessoensis*) IGFBP (PyIGBFP) has been reported to regulate growth [24].

The Pacific white shrimp (*L. vannamei*) is the main marine culture species in the world, and it has important economic values. As a typical crustacean, penaeid shrimp has a distinctive pattern for early development by passing through the embryo, nauplius, zoea, mysis, and postlarvae stages [25]. Meanwhile, shrimp exhibit discontinuous growth through intermittent molting [26]. After every molt, a shrimp can rapidly expand by absorbing water, and there is little or no change in body size until the next ecdysis [27]. However, the detailed mechanisms of growth and development regulation are far from understood. In this study, three LvIGFBP genes were identified from the genome, and the gene structures and expression profiles of these *IGFBP*s were comprehensively analyzed. RNA interference (RNAi) was conducted on *LvIGFBP1* to explore its function on growth. This study provides comprehensive investigations into the structure and function of the IGFBP genes in *L. vannamei* and develops a foundation for understanding the growth mechanism of shrimp.

## 2. Results

### 2.1. Characteristics of IGFBP Sequences

Three IGFBP genes, namely *LvIGFBP1*, *LvIGFBP2*, and *LvIGFBP3*, were identified in the *L. vannamei* genome (Table 1). The lengths of their deduced amino acids were 258, 225, and 109 aa; their molecular weights were 27, 24.3, and 12 kDa; and their isoelectric points (pI) were 4.54, 10.10, and 8.86, respectively.

**Figure 1:**
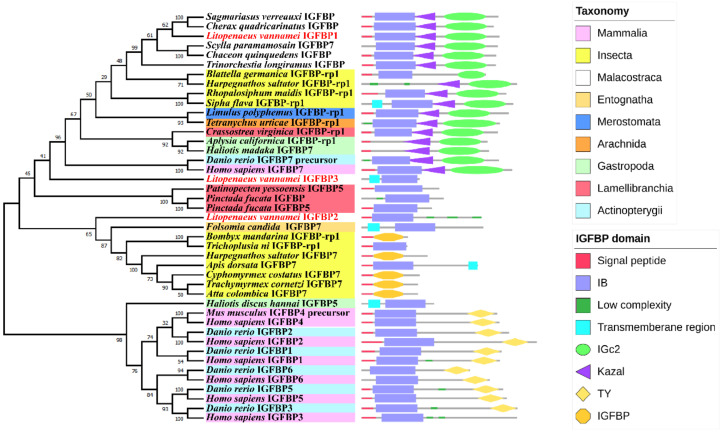
Phylogenetic tree of insulin-like growth factor binding proteins (IGFBPs) and their gene structures. The domains of IGFBPs obtained from the SMART program are located on the right of the phylogenetic tree. The bootstrap values are given at each branch node.

The SMART prediction results showed that only LvIGFBP1 contained three complete domains (the N-terminal IB domain, the mid-region Kazal domain, and the C-terminal IGc2 domain) and showed a high degree of homology to vertebrate IGFBP7 (*Homo sapiens* and *Danio rerio*) and other invertebrate IGFBP/IFGBP-rp1 (Figure 1). Meanwhile, LvIGFBP2 and LvIGFBP3 only contained the N-terminal IB domain. Additionally, LvIGFBP1 and LvIGFBP2 were both found to have a signal peptide at the N-terminal region. LvIGFBP1 had the most complete typical IGFBP structure; its full-length cDNA sequence was found to be 1319 bp, with a 777 bp ORF (Open Reading Frame) encoding a protein of 258 aa.

### 2.2. Phylogenetic Analysis of the IGFBPs

We built a phylogenetic tree using 40 IGFBP superfamily sequences from the NCBI database (Supplementary Table S1) and three IGFBP sequences from *L. vannamei*. The phylogenetic analysis showed that LvIGFBP1, LvIGFBP2, and LvIGFBP3 were clustered into a huge separate branch with IGFBPs from Crustacea; IGFBP-rp1 from Chelicerata (spider mite and horseshoe crab), Hexapoda, and Mollusca; IGFBP7 from *H. sapiens* and *D. rerio*; and IGFBP1–6 from vertebrates form the other branch.

From the phylogenetic tree of IGFBPs, it could be seen that the location of LvIGFBP1 was close to IGFBPs of crustacean and IGFBP-rp1s in various species, and their domains all constituted three typical domains. The large branch containing LvIGFBP1, LvIGFBP2, and LvIGFBP3, we found that the three IGFBPs in shrimp were far from each other—LvIGFBP2 formed a separate cluster with the IGFBP-rp1 or IGBFP7 of Hexapoda, and LvIGFBP3 was located between vertebrate IGFBP7s and bivalve IGFBP5s (Figure 1).

### 2.3. Multiple Sequence Alignment Analyses

**Figure 2:**
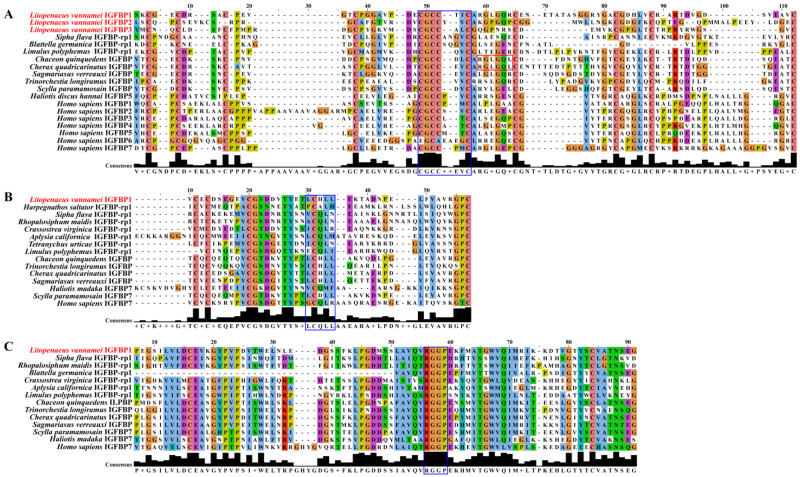
Multiple sequence alignment of the three domains of the IGFBP protein sequence. (**A**) N-terminal insulin growth factor binding (IB) domain: the highly conserved motif is framed in blue (CGCCxxC), except for *Homo sapiens* IGFBP6; the motifs of other species are highly conserved. The conserved decapod motif is framed in red (RxLxxL). (**B**) Mid-region Kazal domain: the conserved motif is framed in blue (LxxLL). This motif is different from other species, and the L site in this motif is not particularly conserved. (**C**) C-terminal immunoglobulin C-2 (IGc2) domain: the highly conserved motif is framed in blue (RGGP). This motif is extremely conserved in most species.

To investigate the amino acid sequence conservation of LvIGFBPs and other IGFBPs, domain sequences were analyzed (Figure 2). Interesting, several conserved motifs have been described as being involved in ligand binding: LxxLL of humans and CGCCxxC of vertebrates and invertebrates were both found to exist in LvIGFBPs. In addition, the conserved RxLxxL motif that exists in decapods is involved in ligand binding as well, but this motif was not found in these three LvIGFBP sequences.

In addition, we performed multi-sequence alignment for the amino acid sequence of the three typical domains. In the IB domain, we found the highly conserved motif CGCCxxC in most species except for IGFBP6 (Figure 2A). It is wort noting that LvIGFBP1–3 have highly conserved Gly amino acid residues that exist in the CGCCxxC motif. In the mid-region Kazal domain, we found the LxxLL motif, but this motif was not as conservative as CGCCxxC in the IB domain. It is noteworthy that the second site of the LxxLL motif is C, and this site is highly conserved (Figure 2B). In the C-terminal of the IGc2 domain, we found a new motif, RGGP, which exists not only in invertebrates but also in some vertebrates, such as *H. sapiens* IGFBP7 (Figure 2C).

### 2.4. Predicted 3D Structure of LvIGFBPs

**Figure 3:**
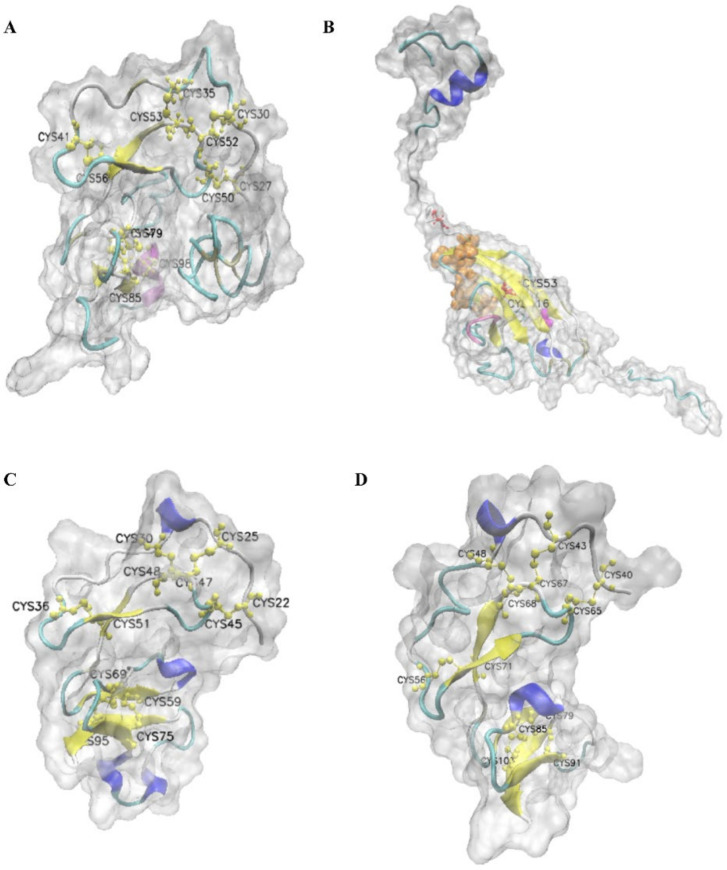
Predicted 3D structure of LvIGFBPs. (**A**) Predicted 3D structure of the N-terminal IB domain of LvIGFBP1. Cysteine residues are in green, and the CGCCxxC motif is in mauve; (**B**) predicted 3D structure of the C-terminal IGc2 domain of LvIGFBP1; (**C**) predicted 3D structure of LvIGFBP2; and (**D**) predicted 3D structure of LvIGFBP3. Cysteine residues are in yellow, predicted ligand binding sites are in orange, predicted enzyme active sites are in magenta, and the RGGP motif is in mauve. The β-sheet is in yellow, the helix is in blue, the β-turn is in cyan, and the random-coil is in white.

From the 3D structure of LvIGFBP1, we found that four β-sheets and one helix exist in the IB domain and six β-sheets and two helixes exist in the IGc2 domain. In the IB domain, there are twelve conserved Cys residues, and these Cys residues comprise six disulfide bonds: C27–C50, C30–C52, C35–C53, C41–C56, C64–C85, and C79–C98. These conserved disulfide bonds stabilize the 3D structure of the protein (Figure 3A). In addition, we found that the highly conserved motif of the IB domain CGCCxxC is located in β-sheet and β-turn. The N-terminal domain of LvIGFBP1 consisted of six disulfide bonds, and the highly conserved CGCCxxC motif formed the front four disulfide bonds to contribute to a rigid, ladder-like subdomain.

Then we made 3D structure prediction for the remaining IGc2 domain of the LvIGFBP1 sequence. The results showed that there were two conserved Cys residues in the IGc2 domain, which formed one disulfide bond, C53–C116; the RGGP motif was located in β-sheet and β-turn. In the C-terminal domain, a relatively large hydrophobic IGF binding surface could contribute to high-affinity IGF binding [28]. Therefore, the C-terminal IGc2 domain may also possess an IGF/ILP binding function. The predicted ligand binding sites of IGc2 were A31, G57, D62, T64, T119, N120, S121, and G123. There is an enzyme active site, G112, in the IGc2 domain (Figure 3B).

By comparison, LvIGFBP2 and LvIGFBP3 both have twelve Cys residues, and they comprise six stable disulfide bonds. In addition, they have five β-sheets. However, they have different helix numbers. LvIGFBP2 has four helixes, and LvIGFBP3 has only two helixes (Figure 3C,D).

### 2.5. Expression Profile of Three LvIGFBPs

**Figure 4:**
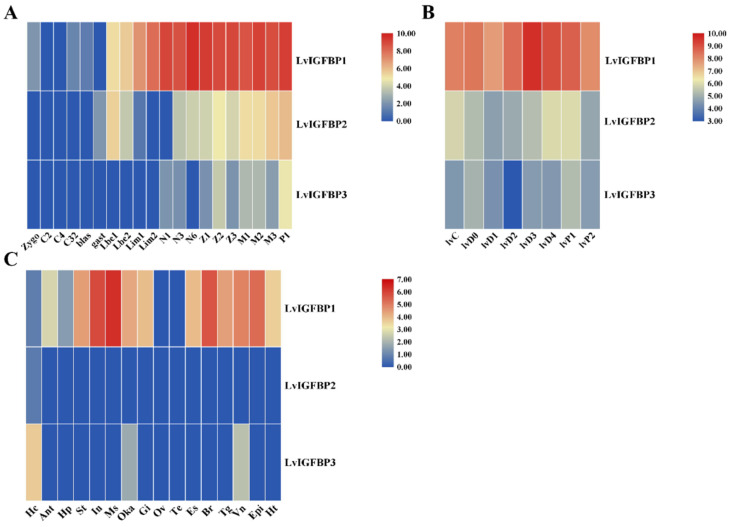
Heatmap of LvIGFBP gene expression profiles in (**A**) early development stages: zygote (zygo), 2 cell (C2), 4 cell (C4), 32 cell (C32), blastula (blas), gastrula (gast), limb bud embryo I (Lbe1), limb bud embryo II (Lbe2), larva in membrane I (Lim1), larva in membrane II (Lim1), nauplius I (N1), nauplius III (N3), nauplius VI (N6), zoea I (Z1), zoea II (Z2), zoea III (Z3), mysis I (M1), mysis II (M2), mysis III (M3), and postlarvae 1 (P1); (**B**) molting stages: the inter-molt (C), pre-molt (D0, D1, D2, D3, and D4), and post-molt (P1 and P2) stages; and (**C**) adult tissues: hemocyte (Hc), antenna (Ant), muscle (Ms), intestines (In), ovary (Ov), stomach (St), lymphoid organ (Oka), gill (Gi), hepatopancreas (Hp), testis (Te), eye-stalk (Es), brain (Br), thoracic ganglion (Tg), ventral nerve (Vn), epidermis (Epi), and heart (Ht).

We analyzed the expressions of three LvIGFBP genes at different development stages and found that *LvIGFBP1* was initially expressed from the zygote to P1 stages. However, *LvIGFBP2* was only detected from the gast to P1 stages, and *LvIGFBP3* was detected from the N1 to P1 stages. *LvIGFBP1* was highly expressed from the Lim1 to P1 stages, and *LvIGFBP2* was highly expressed from the M1 to P1 stages. *LvIGFBP3* was highly expressed at the P1 stage, but its expression level was much lower than *LvIGFBP1* (Figure 4A).

In general, the molting cycle of shrimp can be subdivided into eight recurrent stages: the inter-molt (C), pre-molt (D0, D1, D2, D3, and D4), and post-molt (P1 and P2) stages. *LvIGFBP1* was highly expressed during the entire molting process and peaked at the D3 stage. However, the expressions of *LvIGFBP2* and *LvIGFBP3* were quite low during the molting process (Figure 4B).

To determine the spatial expression of LvIGFBP genes, we analyzed the transcriptional profiles of 16 tissues of *L. vannamei*. Overall, *LvIGFBP2* and *LvIGFBP3* were expressed at low levels (RPKM < 1) in different tissues, though *LvIGFBP3* was highly expressed in the hemocyte. *LvIGFBP1* was highly expressed in most tissues, except for the gonads and the hemocyte, and it was specifically highly expressed in the muscles, intestines, and nerves (Figure 4C). qRT-PCR was employed to quantify the expression of *LvIGFBP1* in different tissues from adult shrimp. The highest expression level of *LvIGFBP1* was observed in the muscles; this result was basically consistent with the expression profile (Supplementary Figure S1).

Therefore, of the three genes, the expression of *LvIGFBP1* is the most significant and was therefore considered for further study.

### 2.6. LvIGFBP1 Gene RNA Interference

**Figure 5:**
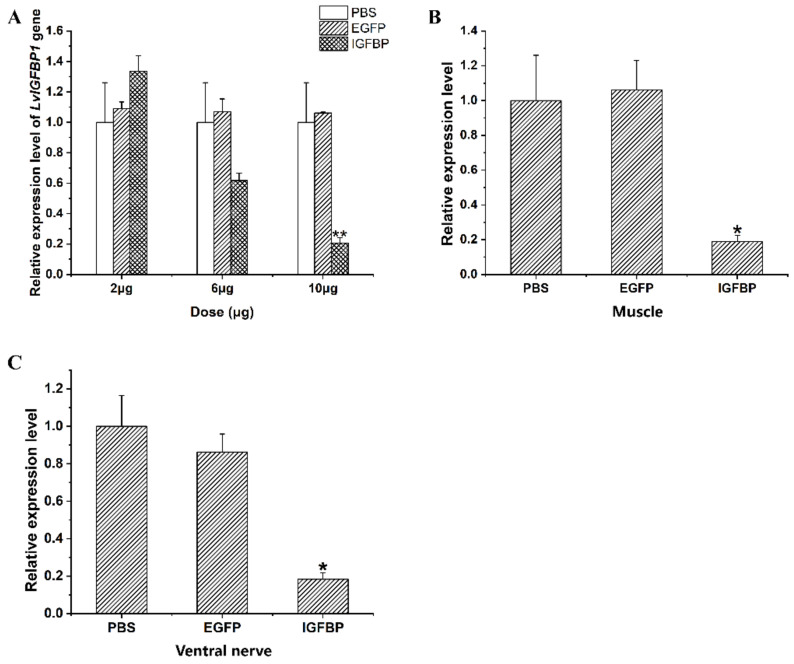
The results of real-time quantitative PCR. (**A**) The silencing efficiency of *LvIGFBP1* RNAi (RNA interference) with different dosages of double stranded RNA (dsRNA). (**B**) Relative expression level of *LvIGFBP1* after RNAi in muscle. (**C**) Relative expression level of *LvIGFBP1* after RNAi in ventral nerve. The expression of target genes was detected by qRT-PCR and normalized to the 18S rRNA gene as the internal reference. These results were based on three independent biological replications and are shown as mean values ± SD. Significant differences of the gene expression levels between three treatments are shown as ** *p* < 0.01 and * *p* < 0.05.

In the pilot experiment of RNA interference, the silencing efficiency of double stranded(ds) *IGFBP1* depended on the dosages of dsRNA injected into the shrimp. The expression level of *LvIGFBP1* gradually decreased with the increase of the ds*IGFBP1* dosage. Compared to the control groups, the expression level of *LvIGFBP1* showed an extremely significant decrease when the dosage of ds*IGFBP1* injected into individuals was 10 μg (Figure 5A). When the dsRNA injection experiment continued for two weeks in *L. vannamei*, the ds*IGFBP1* significantly inhibited the expression levels of *LvIGFBP1* in the muscle and the ventral nerve (Figure 5B,C).

**Figure 6:**
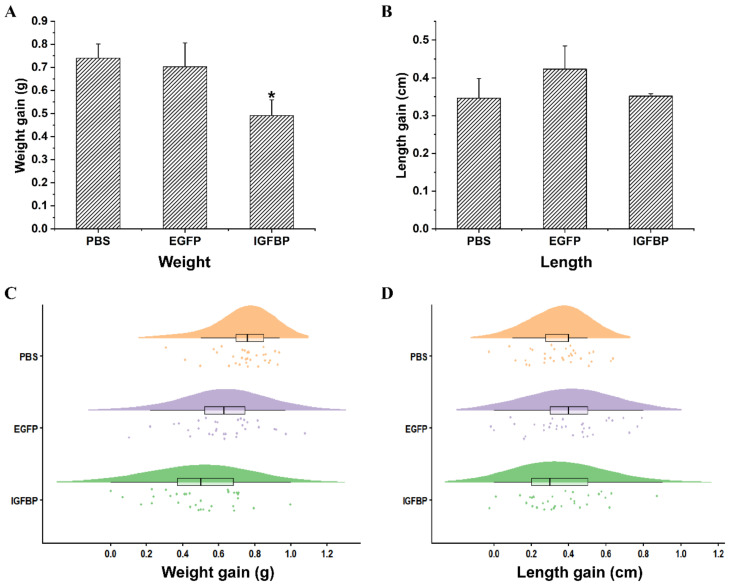
The growth characters after *LvIGFBP1* RNAi in *L. vannamei*. The results were based on three independent biological replications and are shown as mean values ± SD. (**A**) The increase in body weight after *LvIGFBP1* RNAi. (**B**) The increase in body length after *LvIGFBP1* RNAi. (**C**) The raincloud figure reflected the increase in body weight after RNAi. (**D**) The raincloud figure reflected the increase in body length after RNAi. Significant differences of the gene expression levels between three treatments are shown as * *p* < 0.05.

After two weeks of the RNAi experiment, the increment of body weight in the experimental group (ds*IGFBP* group) was significantly lower than the two control groups (phosphate buffer saline, PBS group and double stranded Enhanced green fluorescent protein gene, ds*EGFP* group, *p* < 0.05) (Figure 6A,C). However, while the increment of body length in the experimental group was a little bit lower than that of the ds*EGFP* control group and similar to that of the PBS control group, these results were not significant (*p* > 0.05) (Figure 6B,D).

## 3. Discussion

### 3.1. Structure Characteristics of LvIGFBP Genes

In this study, we analyzed the structure of three LvIGFBP genes from the *L. vannamei* genome. The isoelectric point of LvIGFBP1 is 4.54, so LvIGFBP1 is an acidic protein. In vertebrates, IGFBP1 and IGFBP4 are acidic proteins, and the other IGFBPs (IGFBP2, 3, 5, and 6) are basic proteins. However, in invertebrates, such as *S. verreauxi*, *Blattella germanica*, and *C. quadricarinatus*, the IGFBPs are all acidic proteins.

On the contrary, LvIGFBP2 and LvIGFBP3 contain more basic amino acids and are basic proteins. Domain prediction showed that only LvIGFBP1 contained the mid-region Kazal domain and the C-terminal IGc2 domain, and the other two only contained the N-terminal IB domain. By checking the transcriptome and genome sequences, we confirmed that LvIGFBP2 and LvIGFBP3 genes lack the latter two domains, though not due to incomplete annotations. Combined with amino acid composition and the prediction results of pI, we found that the IB and Kazal domains were sometimes alkaline and were sometimes acidic, though the IGc2 domain was found to be steadily acidic. The pIs of different domains were analyzed, and we found that the pIs of different proteins from invertebrates were mainly dependent on the IB domain, but in vertebrates, the thyroglobulin type I repeat (TY) is the primary determinant of the pI. If the TY domain does not exist, the pI is determined by the IB domain, but the *H. sapiens* IGFBP4 is an exception. Interestingly, most IGFBP7s and IGFBP-rp1s from invertebrates were found to be acidic, except LvIGFBP2, LvIGFBP3, *Harpegnathos saltator* IGFBP7, and *Pinctada fucata* IGFBP and IGFBP5. Due to their acid–base properties and the location of the phylogenetic tree, we guess that the functions of LvIGFBP2 and LvIGFBP3 could be close to that of IGFBP1–6 from vertebrates, and the function of LvIGFBP1 could be close to those of IGFBP7 and IGFBP-rp1.

In terms of the structure of IGFBP proteins, LvIGFBP1 shares the conserved IB domain, a mid-region Kazal domain (which contains the cleavage sites for specific proteinase), and a C-terminal IGc2 domain with other IGFBPs in invertebrates. However, we cannot arbitrarily conclude that LvIGFBPs belong to the IGFBP type instead the IGFBP-rp superfamily because the IGFBP-rp sequence of *Amblyomma americanum* was subsequently found to contain the IGc2 domain in the C terminal. In *H. sapiens* and *D. rerio*, the TY domain exists in the C terminal and has certain specificity and conservativity. Though the C-terminal domains of these sequences vary, the N-terminal domains are conservative.

The N-terminal IB domain is one of the highest conservations in all IGFBPs [10]. Though the molecular weights of LvIGFBP2 and LvIGFBP3 are small, they share a common IB domain organization. By analyzing IGFBP gene structure, twelve conserved cysteines have been found in the N-terminal IB domain, but IGFBP6 is a particular one that only has ten conserved cysteines and six cysteines in the C-terminal domain [10]. We found twelve cysteines in the N-terminal IB domains of all LvIGFBPs, so we could exclude the possibility that LvIGFBPs are IGFBP6. In the N-terminal domain, disulfide bonds are conserved in all IGFBPs and could stabilize the structure of the high-affinity IGF binding subdomain, which contains a compact three-stranded β-sheet [10,12,29]. Thus, the N-terminal IB domain of LvIGFBP1 might bind to IGF/ILP with a high affinity. There are only two conserved cysteines in the C-terminal IGc2 domain of LvIGFBP1, which is quite different to vertebrates. Chernausek and Wetterau found that the high-affinity site is located in the N-terminal domain, and the high-affinity site is sometimes also located in the C-terminal domain [30,31]; thus, the IB domain and the IGc2 domain mainly serve as a binding sites of IGFs. The mid-region Kazal domain is a variable region that contains most of the cleavage sites for specific proteases [30]. Referring to the 3D structure, we believe that there is an G112 enzyme active site in the IGc2 domain (Figure 3B). The function of this site still needs to be further verified by experiments.

In addition, we analyzed the motifs of the three domains. Vertebrate IGFBPs have eighteen conserved cysteines, except for IGFBP6 [32], but LvIGFBPs have nineteen cysteines. The number of conserved cysteines at the N terminal of LvIGFBPs is the same as that of IGFBP1–5, both of which contain twelve cysteines, form six disulfides, and share a conserved CGCCxxC motif [33]. However, there are six cysteines at the C terminal in vertebrate IGFBPs that form three disulfides, while LvIGFBP1 only shows two conserved cysteines and forms one disulfide at the C terminal. In general, the C-terminal domain of IGFBP1–6 mainly belongs to the TY superfamily, while the C-terminal domain of LvIGFBP1 belongs to the immunoglobulin (Ig) superfamily [34]; they have different numbers of conserved cysteines because of the category of their C terminal. The thyroglobulin type I domain may be involved in the inhibitory activity of IGFBPs [35], and IGc2 may be involved in development and the immune system [23,34]. The C-terminal IGc2 domain of IGFBPs also contributes to high-affinity IGF binding, the modulation of IGF actions, and the conferring of some IGF-independent properties [28]. The Ig-like domains are involved in a variety of processes, including cell–cell recognition, cell-surface receptors, and the immune system, and the C-terminal IGc2 domain of LvIGFBP1 may serve as a binding site [11]. Unlike vertebrate IGFBPs, the remaining conserved cysteines of shrimp IGFBPs are all in the mid region. This difference may be related to the functional differences between LvIGFBPs and vertebrate IGFBPs. The C terminals of IGFBP3, 5, and 6 have heparin-binging domains (HBDs: XBBXBX or XBBBXXBX, where B represents a basic amino acid and X represents other amino acids). The heparin-binding domains are used as IGF binding sites, and IGFBP1 uses its C-terminal RGD (Arg-Gly-Asp) sequence as a binding site [32]. In fact, these two motifs do not exist in the C terminal of IGFBPs from invertebrates, but we found a conserved RGGP motif, the specific function of which was not known.

### 3.2. Phylogeny Tree of LvIGFBPs

In the phylogenetic tree, we found that LvIGFBP1 was located in the same branch with IGFBPs from Malacostraca and IGFBP-rp1s from Insecta and Chelicerata. Additionally, these sequences both had highly conserved structures. According to the conclusion of Hwa et al. [34], we believe that LvIGFBP1 belongs to IGFBP-rp1 type, while LvIGFBP2 and LvIGFBP3 may belong to a new IGFBP-rp type, because their mid-region and C-terminal domains are both absent. Hwa et al. reported that the N-terminal domain is ancient [34], and the Kazal and TY domains were also found in cnidarians. Thus, we speculate that LvIGFBP2 and LvIGFBP3 may be have lost domains beyond the N terminal during the evolution process.

### 3.3. Possible Functions of LvIGFBPs

In vertebrates, IGFBPs have multiple functions; they not only regulate IGF activity and bioavailability but also mediate IGF-independent actions, including the inhibition or enhancement of cell growth and the induction of apoptosis [34]. However, there has been a lack of related studies in invertebrates. LvIGFBPs have the N-terminal IB domain, and they may combine with free insulin-like molecules (e.g., ILPs in shrimp) or form a ternary complex with an acid-labile subunit (ALS). Because most circulating IGFs combine with ALS and IGFBP-3 or IGFBP-5 to form a ternary complex in vertebrates, this complex cannot cross the capillary endothelium, thus influencing IGF half-lives and bioavailability [36,37]. In a similar way, the half-life of insulin-like molecules may be prolonged, thus regulating the growth of shrimp.

The results of the RNA interference and qRT-PCR experiments showed that the body weight increment of the dsIGFBP1 group was significantly lower than the PBS and EGFP control groups. When combined with the qRT-PCR results, this revealed that LvIGBFP1 may be a critical factor for the growth of the *L. vannamei*, and the silencing of *LvIGFBP1* can inhibit the growth of shrimp. From this point, our results were consistent with work on the Yesso scallop (*P. yessoensis*), in which *PyIGBFP* is believed to regulate growth [24]. On the other hand, the body length increment of the *LvIGFBP1* RNAi group was not significant, which indicated that LvIGFBP1 may not be directly related to the ecdysone pathway.

IGFBP is known to bind to IGF or ILP in vivo to form complexes, thus extending the half-life of IGF/ILP in vivo. Thus, LvIGBFP1 may bind to ILPs and accumulate a large amount of ILPs to form an ILP pool. Ultimately, LvIGFBP1 may promote the growth of shrimp by increasing the half-life of ILPs. Besides this, from the expression profile of three *LvIGFBP*s, we found that *LvIGFBP3* was highly expressed in hemocytes and rarely expressed in other tissues, and we preliminarily speculated that its function may be to transport ILPs in the hemolymph. Additionally, though *LvIGFBP2* had a low expression in most tissues, different development stages, and different molting stages, it may work during a specific period or with a special external stimulus. Further studies on the gene structure and expression of *LvIGFBP*s will help us understand their functions.

## 4. Materials and Methods

### 4.1. Experimental Animals and Ethical Statement

The experimental shrimp in the postlarve stage were obtained from Guangtai Marine Breeding Company in Hainan province, China, and cultured in the aquarium building of the laboratory of Institute of Oceanology, Chinese Academy of Sciences (Qingdao, Shandong, China). All animal experimentation in this study was conducted in accordance with accepted standards of humane animal care. There were no endangered or protected species applied in these experiments.

### 4.2. IGFBP Sequences Analyses

To identify IGFBP genes, all sequences annotated as IGFBP were extracted from the genome and transcriptome data previously obtained by our laboratory [25,26,27,38]. On the other hand, we used the *S. verreauxi IGFBP* as a reference sequence to run local blast in the *L. vannamei* genome. These obtained sequences were compared by BioEdit (https://bioedit.software.informer.com/7.2/), redundant sequences were removed using CAP3 program [39], and a three-step approach was used to analyze these sequences. Firstly, the *IGFBP* cDNA sequences were submitted to ORF Finder (https://www.ncbi.nlm.nih.gov/orffinder/) and the ExPASy translate tool (http://web.expasy.org/translate/) to obtain deduced amino acid sequences. Secondly, all translated sequences were submitted to SMART (http://smart.embl-heidelberg.de/) to confirm the conserved functional domains of each sequence. Then, the LvIGFBP1 protein sequence was submitted to I-TASSER (https://zhanglab.ccmb.med.umich.edu/I-TASSER/) to predict 3D structure. The result was used with SAVES v5.0 (https://servicesn.mbi.ucla.edu/SAVES/) for protein quality assessment. Finally, validated *IGFBP* sequences were analyzed for phylogeny, 3D structure, and gene expression.

### 4.3. Phylogenetic Analysis

Forty IGFBP orthologous sequences of other species were collected from the NCBI protein database; fourteen of them were from vertebrates (*H. sapiens*, *Mus musculus,* and *D. rerio*), and the others were from invertebrates. Those invertebrate species included four decapods (*S. verreauxi*, *C. quauricarinatus*, *S. paramamosain*, *Chaceon quinquedens*), one amphipod (*Trinorchestia longiramus*), two Lepidoptera (*Bombyx mandarina*, *Trichoplusia ni*), five Hymenoptera (*H. saltator*, *Apis dorsata*, *Cyphomyrmex costatus*, *Trachymyrmex cornetzi*, *Atta colombica*), two Homoptera (*Sipha flava*, *Rhopalosiphum maidis*), one Blattaria (*B. germanica*), one Collembola (*Folsomia candida*), one Xiphosura (*Limulus polyphemus*), one Acarina (*Tetranychus urticae*), two Archaeogastropoda (*Haliotis madaka*, *Haliotis discus hannai*), one Nudibranchia (*Aplysia californica*), two Pterioida (*P. fucata*, *P. yessoensis*), and one Anisomyaria (*Crassostrea virginica*). MEGA X (https://www.megasoftware.net/) served as the main platform that built the phylogenetic tree of these sequences. These IGFBP sequences were aligned by the ClustalW algorithm in the default-model. Then, the phylogenetic tree was constructed by the neighbor-joining (NJ) distance algorithm with a default model. Finally, the mature tree was visualized and ornamented by the Interactive Tree of Life (iTOL) (https://itol.embl.de/) online tool.

### 4.4. Gene Expression Profile

Our laboratory previously conducted DGE (digital gene expression) profiling sequencing for 20 different larval stages, 8 different molting stages, and RNA-Seq analyses for different adult tissues of *L. vannamei* to obtain the FPKM (fragments per kilo bases per million reads) values of each UniGene [25,27]. The FPKM values of the IGFBP genes of *L. vanname* were calculated using the RSEM (version 1.3.0) software (Bo Li, Harvard University, Cambridge, MA, USA) by mapping clean reads to assembled UniGenes from the transcriptome data. The FPKM values were used to compare the difference in expression of all UniGenes, and we chose those with *p* ≤ 0.01 and an absolute value of fold change ≥2 as differentially expressed genes. We conducted log^2^ conversion to normalize data, and then we used these data to draw heatmaps with TBtools (https://github.com/CJ-Chen/TBtools).

### 4.5. In Vivo RNAi Treatment

A pair of specific primers with the T7 promoter sequence, dsIGFBP-F and dsIGFBP-R, were designed to amplify a 609-bp cDNA fragment of the *LvIGFBP1* gene. The PCR procedure was as follows: one cycle of predegeneration at 95 °C for 4 min, 40 cycles of denaturation at 94 °C for 30 s, annealing at 60 °C for 30 s, and one cycle of extension at 72 °C for 10 min. A pair of primers of dsEGFP-F and dsEGFP-R with the T7 promoter sequences were used to clone a 289-bp DNA fragment of the EGFP gene based on EGFP plasmid for double-stranded RNA (dsRNA) synthesis. The PCR procedure was as follows: one cycle of predegeneration at 95 ℃ for 4 min, 40 cycles of denaturation at 94 ℃ for 30 s, annealing at 60 ℃ for 30 s, and one cycle of extension at 72 ℃ for 10 min. The final PCR products were directly purified by a MiniBEST DNA fragment purification kit (TaKaRa, Kyoto, Japan). The purified products served as the template of dsRNA synthesis, and the dsRNA was synthesized and digested by the Transcript Aid T7 High Yield kit (Thermo Fisher Scientific, Waltham, MA, USA) and RNaseA (Thermo Fisher Scientific, Waltham, MA, USA), respectively. Finally, 1.5% agarose gel electrophoresis and a Nanodrop 2000 (Thermo Fisher Scientific, Waltham, MA, USA), were used to detect the quality and the concentration of dsRNAs. The dsRNAs were stored at −80 ℃ until use.

To optimize the silencing efficiency of ds*IGFBP*, we chose 84 shrimp (~2–3 g) to optimize the dsRNA dosage. We set three different injection dosages groups of 2, 6 and 10 μg. Those shrimp were divided into seven groups of 12 individuals each, including three ds*IGFBP* groups (ds*IGFBP*-2 μg, ds*IGFBP*-6 μg, and ds*IGFBP*-10 μg), three ds*EGFP* groups (ds*EGFP*-2 μg, ds*EGFP*-6 μg, and ds*EGFP*-10 μg), and one PBS group. Each group contained three replicates, with 4 individuals in each. Next, we measured the RNAi efficiency of each dsRNA dosage, and 10 μg was chosen to be injected into each shrimp in subsequent RNAi experiments. A total of 108 individuals with body weights of 3.2 ± 0.7 g were divided into three groups (experimental group and the ds*EGFP* and PBS control groups). In terms the ds*EGFP* group, each shrimp was injected with 10 μg of ds*EGFP*, and for the PBS group, each shrimp was only injected with 10 μL of PBS. In terms of the experimental group, each individual was injected with 10 μg of ds*IGFBP* every four days. The experiment lasted for two weeks.

### 4.6. RNA Isolation and cDNA Synthesis

Total RNAs were isolated from different tissues using a Trizol reagent kit (TaKaRa, Kyoto, Japan) according to the manufacturer’s instructions. Agarose gel (1%) electrophoresis and a Nanodrop 2000 (Thermo Fisher Scientific, Waltham, MA, USA) were used to detect the quality and the concentration of RNA, respectively. Then, the first-strand cDNA was synthesized by reverse transcription PCR with 1.5 μg of RNA using the PrimeScript First Stand cDNA Synthesis Kit (TaKaRa, Kyoto, Japan). To eliminate genomic DNA, in the first step, we used a 5× genomic DNA eraser buffer at 42 ℃ for 5 min. The procedure of cDNA synthesis was as follows: 37 ℃ for 1 h and 85 ℃ for 5 s. The cDNA samples were stored at −80 ℃ for further use.

### 4.7. Real-Time Quantitative PCR (qRT-PCR)

To detect the gene expression levels of *LvIGFBP*s in different tissues and after RNAi treatment, we performed SYBR Green-based quantitative real-time PCR. For the reference gene, 18S rRNA was used. The rtIGFBP-F/R and rt18S-F/R primers were used for qRT-PCR amplification. The annealing temperatures of rtIGFBP-F/R and rt18S-F/R were 61 and 55 °C, respectively. The quantitative real-time PCR was run on an Eppendorf Mastercycler ep realplex (Eppendorf, Hamburg, Germany) using SuperReal PreMix Plus (SYBR Green) (Tiangen, Beijing, China) under the following conditions: denaturation at 94 ℃ for 2 min, 40 cycles of 94 ℃ for 20 s, 61 or 55 ℃ for 20 s, and 72 ℃ for 20 s. The comparative cycle threshold (Ct) method with the 2^−∆∆Ct^ equation was used to calculate the relative expression levels of LvIGFBP1 [40].

### 4.8. Statistical Analyses

The statistical significances between controls and different treatments were subjected to one-way ANOVA by using SPSS (version 20) (International Business Machines Corporation, Armonk, NY, USA). The significant difference at *p* < 0.01 was labeled with double asterisks, and that at *p* < 0.05 was labeled with a single asterisk.

## 5. Conclusions

In this study, we identified and characterized three LvIGFBP genes based on genome and transcriptome data. LvIGFBPs possess the conserved domains of the N-terminal IB domain, and they belong to the IGFBP superfamily. The result of *LvIGFBP1* interference demonstrated that it could affect the growth of *L. vannamei*. Our findings provide valuable insight into the structure and function of IGFBP genes in shrimp and crustaceans.

## Figures and Tables

**Table 1:** Characteristics of LvIGFBP genes and predict amino acid sequences.

Gene Name	Deduced Amino Acids	Molecular Weight (kDa)	Isoelectric Point (pI)	Signal Peptide	Accession Number	Location on Genome
*LvIGFBP1*	258	27.03	4.54	1–20	XP_027212706.1	LVANscaffold_1676:698634–704445(−)
*LvIGFBP2*	225	24.35	10.10	1–19	XP_027219293.1	LVANscaffold_1140:490253–502498(−)
*LvIGFBP3*	109	12.00	8.86	-	XP_027214055.1	LVANscaffold_1781:46638–49525(−)

## Data Availability

The data presented in this study are available on request from the corresponding author.

## Supplementary Materials

The following are available online at https://www.mdpi.com/1422-0067/22/3/1056/s1, Figure S1: Expression of LvIGFBP1 analyzed by real-time PCR in different shrimp tissues, Table S1: NCBI accession numbers for all IGFBP superfamily sequences in phylogenetic analysis.
