# Supplementary material for: Characterization and Expression Analysis of Insulin Growth Factor Binding Proteins (IGFBPs) in Pacific White Shrimp Litopenaeus vannamei
Source: Int J Mol Sci. 2021 Jan 21;22(3):1056. doi: 10.3390/ijms22031056 (PMC7866140; doi:10.3390/ijms22031056)
Supplement: Supplementary file 1 [file ijms-22-01056-s001.pdf]

Supplementary Materials

Characterization and Expression Analysis of Insulin Growth Factor Binding Proteins (IGFBPs) in Pacific White Shrimp *Litopenaeus vannamei*

Ying Pang, Xiaojun Zhang, Jianbo Yuan, Xiaoxi Zhang, Jianhai Xiang and Fuhua Li

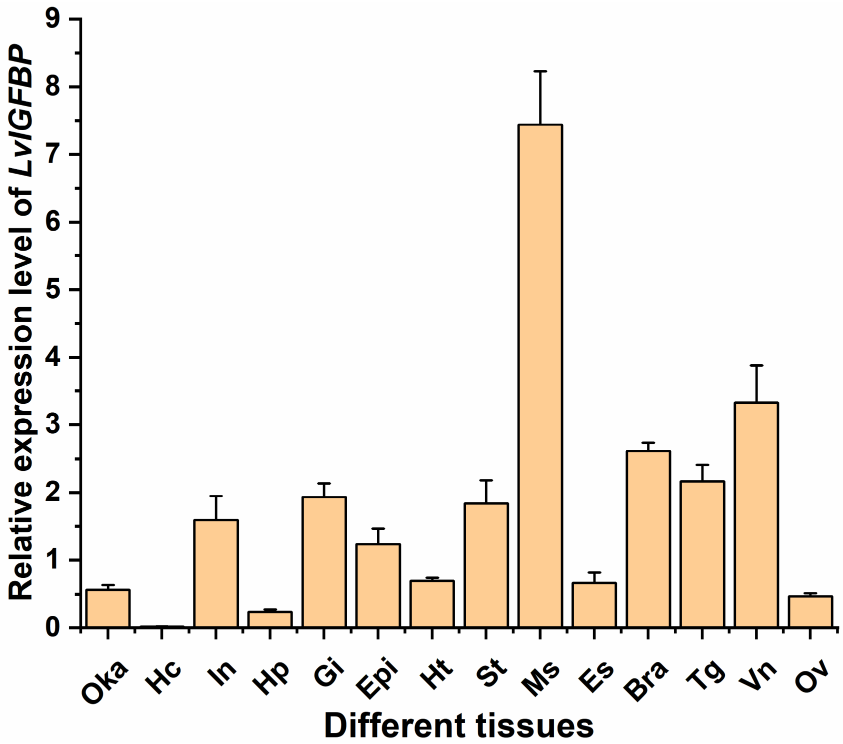

Figure S1. Expression of LvIGFBP1 analyzed by real-time PCR in different shrimp tissues. Abbreviations used in this figure are as follows: Hemocyte (Hc), Muscle (Ms), Intestines (In), Ovary (Ov), Stomach (St), lymphoid organ (Oka), Gill (Gi), Hepatopancreas (Hp), Eye-stalk (Es), Brain (Bra), Thoracic ganglion (Tg), Ventral nerve (Vn), Epidermis (Epi), Heart (Ht). Each bar is means ± SD of three shrimps for different tissue.18S rRNA served as reference gene.

Table S1. NCBI accession numbers for all IGFBP superfamily sequences in phylogenetic analysis.

Species			Gene	pI	Accession Number
**Chordata**					
Mammalia	Primates	*Homo sapiens*	IGFBP1	5.11	AAA52783.1
			IGFBP2	7.48	AAB22308.1
			IGBFP3	9.03	EAL23801.1
			IGBFP4	6.81	AAA62670.1
			IGBFP5	8.58	AAA53505.1
			IGBFP6	8.15	EAW96664.1
			IGBFP7	8.25	AAH66339.1
	Rodentia	*Mus musculus*	IGFBP4 precursor	6.81	NP_034647.1
Actinopterygii	Cypriniformes	*Danio rerio*	IGFBP1	7.82	CAC44453.1
			IGFBP2	6.59	AAF23123.1
			IGFBP3	8.43	CAC44454.1
			IGFBP5	8.66	AAM51549.1
			IGFBP6	9.00	ABV58580.1
			IGFBP7 precursor	7.38	NP_998089.1
**Arthropod**					
Malacostraca	Decapoda	*Sagmariasus verreauxi*	IGFBP	4.71	ALZ50690.1
		Cherax quauricarinatus	IGFBP	4.66	AGS78412.1
		Scylla paramamosain	IGFBP7	4.40	ALO50698.1
		Chaceon quinquedens	IGFBP	4.40	ART33388.1
	Amphipoda	*Trinorchestia longiramus*	IGFBP	5.31	KAF2359210.1
Insecta	Lepidoptera	*Bombyx mandarina*	IGFBP-like1	6.14	XP_028026220.1
		Trichoplusia ni	IGFBP-like1	4.85	XP_026744518.1
	Hymenoptera	*Harpegnathos saltator*	IGFBP-rP1	5.45	XP_011143747.2
			IGFBP7	7.95	EFN79600.1
		Apis dorsata	IGFBP7	4.98	XP_006622917.1
		Cyphomyrmex costatus	IGFBP7	4.45	KYM95970.1
		Trachymyrmex cornetzi	IGFBP7	4.53	KYN08935.1
		Atta colombica	IGFBP7	4.79	KYM88446.1
	Homoptera	*Sipha flava*	IGFBP-rP1	6.42	XP_025409042.1
		Rhopalosiphum maidis	IGFBP-rP1	5.40	XP_026819885.1
	Blattaria	*Blattella germanica*	IGFBP-rP1	5.44	PSN40882.1
Entognatha	Collembola	*Folsomia candida*	IGFBP7	5.27	OXA42780.1
Merostomata	Xiphosura	*Limulus polyphemus*	IGFBP-rP1	4.80	XP_013780174.1
Arachnida	Acarina	*Tetranychus urticae*	IGFBP-rP1	5.21	XP_015793232.1
**Mollusca**					
Gastropoda	Archaeogastropoda	*Haliotis madaka*	IGFBP7	5.98	ALU63746.1
		Haliotis discus hannai	IGFBP5	5.77	QNG71088.1
	Nudibranchia	*Aplysia californica*	IGFBP-rP1	5.66	XP_005097687.1
Lamellibranchia	Pterioida	*Pinctada fucata*	IGFBP	8.38	ANN23501.1
			IGFBP5	8.58	ALJ32264.1
		Patinopecten yessoensis	IGFBP5	8.93	KF801669.1
	Anisomyaria	*Crassostrea virginica*	IGFBP-rP1	5.72	XP_022345586.1
